# Advancements in Gene Therapy for Non-Small Cell Lung Cancer: Current Approaches and Future Prospects

**DOI:** 10.3390/genes16050569

**Published:** 2025-05-12

**Authors:** Iwona Ziółkowska-Suchanek, Natalia Rozwadowska

**Affiliations:** Institute of Human Genetics, Polish Academy of Sciences, Strzeszyńska 32, 60-479 Poznań, Poland; natalia.rozwadowska@igcz.poznan.pl

**Keywords:** gene therapy, NSCLC, CRISPR/Cas9, tumor-suppressor genes, viral vectors, targeted therapies, immune modulation, CAR-T therapy, tumor microenvironment

## Abstract

Non-small cell lung cancer (NSCLC) is the leading cause of cancer-related death worldwide, characterized by late diagnosis and resistance to conventional therapies. Gene therapy has emerged as a promising alternative for NSCLC therapy, especially for patients with advanced disease who have exhausted conventional treatments. This article delved into the current developments in gene therapy for NSCLC, including gene replacement and tumor suppressor gene therapy, gene silencing, CRISPR/Cas9 gene editing, and immune modulation with CAR-T cell therapy. In addition, the challenges and future prospects of gene-based therapies for NSCLC were discussed.

## 1. Introduction

Globally, lung cancer is the second most common cancer and the leading cause of cancer-caused mortality. According to the GLOBOCAN 2020 estimates lung cancer was diagnosed in 2.2 million cancer patients and was a cause of death of 1.8 million patients in 2021 [1]. Lung cancer is the leading cause of cancer incidence and mortality in men, while it ranks third in terms of incidence in women [2]. Non-small cell lung cancer (NSCLC) accounts for approximately 85% of all lung cancer cases, with an overwhelming majority diagnosed at advanced stages where survival rates remain poor. The three main subtypes of NSCLC include: adenocarcinoma (40%), squamous cell carcinoma (25%) and large cell carcinoma (10%). The most common histologic subtype of NSCLC is adenocarcinoma. In clinical medicine, the treatment of NSCLC uses innovative therapeutic approaches, such as targeted therapies or combined therapies. Nevertheless, the survival rate of patients with lung cancer is poor and the prognosis is unfavorable. In most populations, the survival time of patients within 5 years of diagnosis is only 10% to 20% [2]. The relative 5-year survival rate is strongly dependent on the stage of the disease at the time of diagnosis: local (61%), regional (34%) or distant (7%) stage [3]. The prognosis of patients with NSCLC remains poor, as most cases are diagnosed at the metastatic stage with the most common sites of metastasis in the brain (20–40% of patients), bones (39%) and liver (16.3%). NSCLC contributes to approximately 50% of all brain metastases occurred in cancer patients [4]. Late diagnosis of lung cancer is primarily an issue of the lack of recognition of lung cancer symptoms and the long time it takes to complete diagnostic tests. There is a continuing need for effective early detection programs and for raising awareness of the risks associated with cigarette smoking [5]. Despite significant advances in targeted therapies and immunotherapies, resistance mechanisms and heterogeneity within tumors often undermine treatment efficacy. As such, innovative therapeutic strategies are essential, and gene therapy has emerged as a promising modality.

## 2. Molecular Landscape of NSCLC

NSCLC exhibits a complex molecular landscape characterized by various genetic mutations and alterations that influence tumor behavior and treatment responses. The key genetic alternations of NSCLC were summarized in Table 1. Understanding these molecular features is crucial for developing targeted therapies and personalized treatment strategies.

Approximately 50% of NSCLC cases harbor mutations in the *p53* gene [6], with a higher prevalence in squamous cell carcinoma (76.7%) compared to adenocarcinoma (45.6%). The most common oncogenic mutation detected in patients with NSCLC is EGFR activating mutations, observed in 10–20% of Caucasians and more than 50% in the Asian population (data according to COSMIC database) [7]. *EGFR* mutations occur in approximately 25.8% of adenocarcinoma cases, with exon 20 insertions being a significant subtype. These mutations are actionable, and therapies like Osimertinib targeting the ATP- binding site of mutated *EGFR* have shown efficacy [8]. *KRAS* mutations with a frequency of around 32.2% [9] are present in adenocarcinoma cases. The G12C *KRAS* mutation is notable, as it is targetable by specific inhibitors like sotorasib which keep it in inactive state and prevent the downstream effect of its overactivation [10]. *ALK* rearrangements are present in about 5–10% of NSCLC cases, lead to the production of fusion proteins that drive tumorigenesis. *ALK* inhibitors, such as crizotinib, have been developed to target these alterations [11]. *ERBB2* (HER2: human epidermal growth factor 2) alterations are found in approximately 5.4–6% of NSCLC patients [12]. The most common alteration is exon 20 insertion, observed in 58% of cases in a Chinese cohort and 41.6% in a U.S. cohort [13]. These alterations are actionable, with targeted therapies e.g., trastuzumab, deruxtecan, a HER2-targeted antibody-drug conjugate, available [14]. *BRAF* (v-raf murine sarcoma viral oncogene homolog B) gene mutations, particularly V600E, are present in 1–2% of NSCLC cases [8]. Targeted therapies like vemurafenib and dabrafenib have shown efficacy in these cases [15]. Nowadays, molecular landscape of driver mutations in NSCLC comprises genomic alterations in *MET* (receptor tyrosine kinase). *MET* exon 14 mutations were detected in about 3–4% of NSCLC cases and are strongly associated with tumor progression [16]. *MET* inhibitors, such as capmatinib [17], have been developed for treatment. NTRK1 (neurotrophic receptor tyrosine kinase) gene fusions are rare but actionable alterations found in approximately 3% of NSCLC cases [18]. TRK inhibitors like entrectinib and larotrectinib have demonstrated effectiveness [19]. In addition to the key genetic changes mentioned above, changes in the sequence of *c-ROS1* (receptor tyrosine kinase) and *NGR1* (neuregulin-1) genes were reported [20]. The molecular landscape of NSCLC is complex and varies between histological subtypes. Since the aforementioned inhibitors are widely used as targeted gene therapies, these aren’t gene therapy in the classical sense but rather genotype-specific therapies based on molecular profiling. Gene therapy in NSCLC aims to repair mutated oncogenes or tumor suppressor genes, modulate immune responses, and exploit genetic vulnerabilities for targeted treatment. This review focuses on recent developments in gene therapy techniques and their potential clinical applications in NSCLC.

## 3. General Mechanisms of Gene Therapy in NSCLC

**Figure 1:**
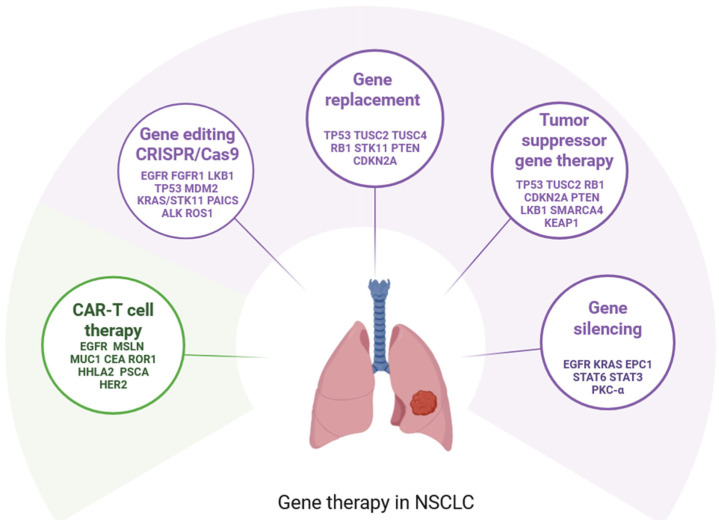
Gene therapy for non-small cell lung cancer with studied target genes. Created in https://BioRender.com.

Gene therapy in NSCLC is a promising but still developing approach that targets the genetic drivers of cancer, rather than relying solely on traditional approaches such as chemotherapy or radiation therapy. The approach involves delivering genetic material into cancer cells or surrounding tissue to alter gene expression and counteract cancer growth. The overarching goal is to correct genetic defects, sensitize tumors to treatments, or induce immune responses. Gene therapy for NSCLC can be broadly categorized into several approaches, each targeting specific molecular mechanisms underlying tumor progression and resistance (Figure 1). Below the major mechanisms of gene therapy used in NSCLC were described.

## 4. Gene Replacement

One of the earliest strategies in gene therapy for NSCLC involved replacing defective or missing tumor-suppressor genes. The *p53* gene, often mutated in NSCLC, is crucial for regulating the cell cycle and initiating apoptosis in response to DNA damage. The *p53* protein has a fusion of apoptotic regulation is associated with selective activation of apoptotic target genes such as *JMY*, *ASPP* and other genes of the *p53* family, *p63* and *p73* [21]. Restoring *p53* function has been shown to suppress tumor growth and enhance chemosensitivity. Adenovirus-mediated *p53* cancer gene therapy develops and proves to be a promising antitumor strategy to restore the wild-type *p53* function. One of the I phase clinical trials explored the use of an adenoviral *p53* vector (Adp53) to 21 patients with advanced NSCLC which produced little toxicity. The treatment was well-tolerated, and some patients showed signs of clinical benefit [22]. Also, one of the first studies conducted by S. G. Swisher et al. also confirmed that repeated intratumoral injections of Ad-p53 were well tolerated and mediated antitumor activity in a subset of patients with advanced NSCLC [23]. Another adenovirus-mediated gene therapy, based on SCH-58500, was designed to deliver the *p53* tumor suppressor gene to NSCLC cancer cells. In subsequent studies, Schuler M. et al. proposed adenoviral *p53* gene therapy in twenty-five patients with nonresectable, advanced NSCLC patients. They observed that intratumoral adenoviral *p53* gene therapy may not provide additional advantage in patients receiving effective first-line chemotherapy for advanced stage of NSCLC [24]. Clinical investigations into SCH-58500 for NSCLC have been limited. Early-phase clinical trials aimed to assess the safety and potential efficacy of this gene therapy approach. However, detailed results from these studies have not been widely published, and SCH-58500 has not become a standard treatment for NSCLC. However the study of B. Deng et al. showed that the wound surface injection of recombinant adenoviral *p53* gene (rAd-p53) has shown effective activity in preventing relapse or metastasis and improving both progression-free survival/overall survival after conventional surgery in patients with NSCLC [25]. To summarize, there are no *p53*-targeted therapies that have received regulatory approval for the treatment of NSCLC. Additionally, a Phase II study evaluated the combination of surgery and adenoviral *p53* gene therapy, suggesting that this approach might improve patient outcomes compared to surgery alone [25]. Ongoing studies are exploring alternative strategies, such as targeting the negative regulators of *p53* to enhance its tumor suppressor function with the use of the MDM2/MDMX inhibitors [26]. Phase I and II clinical trials have demonstrated the potential of adenovirus-based *p53* gene therapy for the treatment of NSCLC, but these therapies are not yet part of standard clinical practice. They remain in the realm of clinical trials, and studies conducted on larger groups of patients are needed to confirm the efficacy of *p53* gene therapy and help establish safety profiles.

Another candidate for NSCLC gene therapy was identified in the 3p21.3 region and its function was described as “gatekeeper” in the molecular pathogenesis of lung cancer [27]. Previous in vitro studies have shown that genes located in the 3p21.3 region exhibit varying degrees of tumor suppression. The *TUSC2* (Tumor Suppressor Candidate 2) gene with the highest tumor suppressor activity is located in the same region [28]. Most of the lung cancer cell lines studied did not show expression of the TUSC2 protein. A genome-wide, unbiased comparative study of tissues from lung adenocarcinoma patients (39 smokers and 30 non-smokers) showed a significant reduction in TUSC2 expression, which was associated with a significantly worse prognosis [28,29]. Large-scale analysis of TUSC2 expression in lung cancer and in metaplastic and dysplastic bronchial lesions found significantly lower levels of TUSC2 relative to normal hyperplastic epithelium. The expression level of *TUSC2* was found to have the strongest proapoptotic activity in NSCLC cells compared to other 3p21.3 tumor-suppressor genes candidates [28,30]. In studies of C. Lu et al., the *TUSC2*-expressing plasmid vector was packaged in DOTAP:cholesterol (DOTAP:chol) nanovesicles developing a gene delivery agent that can be administered intravenously [31]. In the first systemic gene therapy with DOTAP:cholesterol nanoparticles in lung cancer patients, Ch. Lu et al. demonstrated gene uptake by primary and metastatic tumors, confirmed transgene and gene product expression, the occurrence of specific changes in TUSC2-regulated pathways, and anti-tumor effects [30,31].

Additionally, in the region on chromosome 3p21.31 containing the cluster of tumor suppressor genes like *TUSC2/FUS1*, the *NPRL2* (Nitrogen Permease Regulator-Like 2) also known as *TUSC4* was identified [27,32]. Previous studies have shown that the *NPRL2/TUSC4* tumor suppressor gene exhibits reduced expression in NSCLC [33]. Recently, I. Meraz et al. analyzed the anti-tumor immune response of *NPRL2* in NSCLC aPD1R/KRAS/STK11mt in humanized mice [34]. KRAS/STK11mt/aPD1R A549 NSCLC cells were used to generate metastases and were treated with NPRL2 in combination with pembrolizumab. NPRL2 treatment significantly reduced metastasis, while pembrolizumab was ineffective. The anti-tumor effect was correlated with increased infiltration of human cytotoxic T cells and down-regulation of myeloid and regulatory T cells in TME. In addition, it was shown that stable expression of NPRL2 resulted in downregulation of MAPK and AKT-mTOR signaling. *NPRL2* gene therapy was confirmed to induce antitumor activity in KRAS/STK11mt/aPD1R tumors through dendritic cell-mediated antigen presentation and cytotoxic activation of immune cells. This study presents a novel finding on *NPRL2* gene therapy with potential clinical application value [34].

In summary, while many gene-targeted therapies for NSCLC have shown potential in early studies, they are not yet available for clinical use. Various gatekeeper genes have been considered in gene therapy for NSCLC. As with *p53*, one of the major players in gene therapy, several new candidates have been discovered, such as *TUCS2* and *TUSC4*. For mutant gatekeepers of NSCLC, the basis of gene therapy is to restore the wild-type conformation of the gene. Apart from a number of clinical trials, these therapies are still difficult to apply in clinical practice. In the case of p53, it is known that p53 levels remain low in normal cells, so the question is how much p53 should be replaced to achieve the highest efficacy while maintaining safety. Another new approach in gene replacement therapies is to target gatekeeper gene regulatory factors, such as Mdm2/MdmX, to control p53 protein activity. The biggest challenge in the future will be to elucidate the essential interactors of target genes and the mechanisms underlying their anti-tumor activity. This knowledge will reveal the drug resistance of cancer cells, which is caused by the activity of the interactors. In addition, the discovery of new drug delivery methods, such as novel viral vectors combined with new inhibitors such as small-molecule inhibitors, will greatly support standard treatment strategies and may lead to the discovery of combination treatment strategies.

## 5. Gene Silencing

Another gene therapy approach based on gene silencing strategies, including RNA interference (RNAi) and antisense oligonucleotide (ASO) therapies, have been extensively researched as potential treatments for NSCLC. These approaches aim to suppress the expression of oncogenes or other genes critical for tumor growth and survival.

In NSCLC there were several studies which utilized small interfering RNAs (siRNAs). In the study of G. Chen the EGFR, frequently overexpressed or mutated in NSCLC, was targeted. In different cell lines derived from NSCLC, the combined effect of RNA interference targeting the *EGFR* mRNA, and inactivation of *EGFR* signaling using different receptor tyrosine kinase inhibitors (TKIs) or a monoclonal antibody cetuximab were investigated. This approach led to enhanced growth inhibition and induced apoptosis in NSCLC cell lines, regardless of their *EGFR* mutation status. The most significant effect was observed when afatinib, a TKI, was combined with *EGFR*-specific siRNA [35].

Mutations in the *KRAS* gene in NSCLC are associated with poor prognosis. Research combining KRAS-specific siRNAs with miR-34, a tumor-suppressor microRNA, showed reduced tumor growth and increased sensitivity to cisplatin in vitro and in vivo using KRAS-p53 mutated mouse models of lung adenocarcinoma [36]. In the study by L. She et al., miR-30c and miR-21 were significantly up-regulated by both KRAS isoforms and induced drug resistance and increased cell migration and invasion, likely mediated by inhibition of key tumor suppressor genes (*NF1, RASA1, BID, RASSF8*). Moreover, in early-stage NSCLC tumor tissues, the expression of miR-30c and miR-21 was significantly elevated compared to normal lung tissues. The Authors indicated that systemic delivery of LNA-anti-miR-21 combined with cisplatin treatment in vivo completely suppressed the development of tumors in a mouse model of lung cancer [37]. KRAS-driven treatment of NSCLC was implemented with BF006—novel lyophilized lipid NP formulation, which delivers a siRNA inhibiting the expression of glutathione-S-transferase P that is strongly up-regulated in KRAS mutated tumors [38]. The same group indicated that in orthotopic NSCLC tumor model, the survival rate of the NBF-006 treatment group was significantly prolonged compared with the control group which supported development of NBF-006 into clinical studies [38].

Short hairpin RNA (shRNA) approach was used to silencing the enhancer of polycomb 1 (*EPC1*) in NSCLC cell line. This gene is implicated in cancer progression. Silencing *EPC1* in A549 NSCLC cells led to decreased cell proliferation and tumor growth in vitro and in vivo, suggesting *EPC1* as a potential therapeutic target [39].

ASO therapy was also introduced in NSCLC treatment. ASO therapies have been investigated targeting various oncogenes like *STAT6* (Signal transducer and activator of transcription 6) which plays a role in tumor progression and immune evasion. Recently, K. He at al. targeted *STAT6* by ASO approach along with hypofractionated radiotherapy to primary tumors in three bilateral murine NSCLC models (Lewis lung carcinoma, 344SQ-parental, and anti-PD-1-resistant 344SQ lung adenocarcinomas). The Authors found that *STAT6* ASO combined with radiotherapy slowed growth of both primary and abscopal tumors, decreased lung metastases, and extended survival which may be an alternative therapeutic approach for patients with immune-resistant NSCLC [40].

The *STAT3* (signal transducer and activator of transcription 3) was also being considered as a potential drug in the ASO gene therapy approach. Systemic delivery of the unformulated ASO, AZD9150, decreased STAT3 expression among preclinical models (human primary patient-derived tumor explant models, included NSCLC, colorectal cancer, and lymphoma). AZD9150’s antitumor activity was also proven in patients with refractory lymphoma and NSCLC in a phase I dose-escalation study [41]. In study of Ch. Tang et al., the STAT3 ASO activity was checked in phase II clinical trial conducted evaluating combination treatment with danvatirsen (ASO that inhibits STAT3) and durvalumab (anti-PD-L1) in patients NSCLC [42]. Since the majority of subjects exceeded the predetermined threshold of zero 4-month disease control rate, the selected combination was considered promising. Further, in vitro studies revealed an anti-inflammatory and pro-tumour effect of *STAT3* ASO. In addition, preclinical evidence suggests that *STAT3* ASO can enhance myeloid and fibroblast suppressive function, rather than solely inhibit STAT3 function. The above studies have indicated the need for future studies using STAT3 inhibition, which may achieve greater efficacy if appropriate combinations are developed or the drug is adjusted to reduce the bone marrow-derived immunosuppressive effects [42].

Protein Kinase C-alpha (*PKC-α*) has been investigated as a therapeutic target in NSCLC through the use of ASOs, particularly aprinocarsen. Preclinical studies demonstrated that aprinocarsen (LY900003) could inhibit *PKC-α* expression, leading to reduced tumor growth in NSCLC models. The ASO LY900003 (ISIS 3521) inhibits *PKC-α* expression through RNase H-mediated cleavage of the hybridized *PKC-α* mRNA. In preclinical studies, aprinocarsen showed inhibition of the *PKC-α* mRNA and reduced production of PKC-α protein in the A549 NSCLC cell line [43]. Early-phase clinical trials demonstrated that LY900003 was well-tolerated and could be feasibly combined with chemotherapy. LY900003 can be administered safely in combination with cisplatin and gemcitabine and is linked to antitumor activity in patients with advanced NSCLC (stage III and IV) [44]. However, further studies are needed to establish its efficacy because further clinical trials yielded mixed results. A phase II study combining aprinocarsen with gemcitabine and carboplatin showed moderate activity but resulted in significant toxicities, including severe thrombocytopenia [45,46]. Subsequent phase III trials did not demonstrate a survival benefit when aprinocarsen was added to standard chemotherapy regimens [47]. These findings suggest that while targeting PKC-α with ASOs like aprinocarsen showed promise in preclinical models, the approach did not translate into clinical benefit for NSCLC patients. As a result, research efforts have shifted towards exploring other molecular targets and therapeutic strategies in NSCLC treatment.

## 6. Gene Editing: CRISPR/Cas9 in NSCLC

The CRISPR/Cas9 technique is an excellent tool not only for modulating the activity of genes important in the etiopathogenesis of NSCLC, but also offers the possibility of using screening to study genetic dependencies and potential therapeutic targets in NSCLC. In addition, the technique has been used to understand the causes of drug resistance in NSCLC and to create unique research models of lung cancer.

CRISPR-Cas9 technology has emerged as a promising approach to target protooncogenes and tumor suppressor genes altered in NSCLC as well as anticancer drug resistance in NSCLC. It appears that precise genome editing of cancer cells using CRISPR/Cas9 can disrupt key genes that determine resistance or sensitize cancer cells to existing therapies. In the study of T. Koo, a single nucleotide missense mutation (CTG > CGG) in exon 21 of *EGFR* which results in one of the major *EGFR* activation mutations (L858R) in NSCLC was targeted [48]. The L858R mutation, accounts for approximately 43% of *EGFR*-mutated lung adenocarcinoma. This transversion led to kinase domain activation and increased signal transduction to the downstream pro-survival pathways. To investigate oncogenic mutant allele-specific cleavage mediated by CRISPR/Cas9, they used xenograft mice implanted separately with two NSCLC cell line (H1975, A549). The disruption of the *EGFR* mutant allele was essential for NSCLC cell killing effect which indicated that the mutant-specific Cas9 allele can effectively differentiate the mutant *EGFR* allele from the wild-type allele, leading to targeted disruption of the oncogene and cancer cell death. In addition in murine xenograft model of human lung adenocarcinoma the Authors confirmed that Ad-mediated *EGFR* oncogene-specific Cas9 expression can disrupt the *EGFR* mutant allele in H1975 cells with high accuracy in vivo [48]. In subsequent studies of A. Cheung et al. designed an anti-L858R targeting carrier that contained Cas9 nuclease and gRNA specific for L858R. The obtained results confirmed the role of driver mutation L858R in promoting tumor proliferation in cancer cells, with consequences of CRISPR/Cas9-mediated knockout demonstrated at the DNA, protein, and functional levels in the mutant cells compared to wild type cells [49].

To identify the genetic determinants underlying sensitivity to *FGFR*-targeted therapy, Z. Yang et al. performed a CRISPR/Cas9 kinome (human protein kinases) knockout in *FGFR1*-enhanced lung cancer cells [50]. The results of this study revealed essential genes for NSCLC with a preponderance of kinase genes, including *CSNK2A1* (*CK2α*), *PIP4K2C* (*PIP4kγ*) and *PLK1*. The *PLK1* gene was unraveled as a previously underappreciated therapeutic target whose loss of function is synthetically lethal with *FGFR*-targeted therapy in lung cancer with *FGFR1* amplification [50].

In study of M. Severino et al. used the CRISPR/Cas9 system to generate cell lines with a CRISPR-edited LKB1 isoform (called Super LKB1) [51]. The *STK11* gene is one of the main regulators of cellular metabolism through activation of *AMPK* under nutrient-deficient conditions. The authors observed that cisplatin was more effective in Super LKB1+ cells, suggesting that the absence of *LKB1* and high *NRF2* activity in A549 WT may promote resistance to oxidative damage by cisplatin. The potential use of *STK11* gene editing by NHEJ-CRISPR to restore LKB1 expression, and consequently cellular signaling of the AMPK pathway, was confirmed [51].

As mentioned in the introduction to this section, the CRISPR/CAS9 technique allows screening for genetic dependencies and potential therapeutic targets in NSCLC. Q. Wang et al. conducted genome-wide CRISPR/Cas9 screening in two NSCLC cell lines (NCI-H460 and A549) containing *p53* wild-type and receptor tyrosine kinase (wt p53-RTK) genes using the GeCKO v2.0 lentiviral library (comprising 123,411 small guide RNAs [sgRNAs] targeting 19,050 human genes) [52]. Bioinformatics analysis based on TCGA data showed that the *MDM2* gene was highly expressed in p53 wild-type NSCLC compared to p53 mutant NSCLC, implying that *MDM2* overexpression may be beneficial for *p53* wild-type cancer growth. *MDM2* is previously known oncogene that negatively regulates *p53* by its E3 ubiquitin ligase function. Further studies showed that *MDM2* knockout led to reduced cell proliferation and tumor growth. The anti-tumor effect of pharmacological MDM2 inhibition was tested using a selective small-molecule inhibitor (RG7388) which inhibited cell proliferation through apoptosis induction and cell cycle blockade, and inhibited wt p53-RTK NSCLC tumor growth in xenograft models. It has been shown that pemetrexed can increase the expression of the p21 protein of the p53-MDM2 pathway, so it was selected for combination treatment with RG7388 on NSCLC cell lines. The results showed synergistic inhibitory effects of RG7388 and pemetrexed on wt p53-RTK NSCLC cells by enhancing apoptosis and anti-tumor effects [52]. The authors introduced the *MDM2* gene as a new therapeutic option for NSCLC patients with wild-type *p53* and RTK who are unsuitable for currently available targeted therapies.

In another study, S. Mukhopadhyay used genome-wide CRISPR/Cas9 screening to identify genes whose inactivation enhances the efficacy of two drugs (MRTX-849: adagrasib, *KRAS* G12C Inhibitor; TNO-155: SHP2i, inhibitor of wild-type *SHP2*) in four NSCLC cell lines with *KRAS/STK11*-mutant NSCLC lines [53]. Screening identified potentially targeted synthetic lethal (SL) genes, including serine/threonine kinases, enzymes that modify tRNA and proteoglycan synthesis, and components of the *YAP*/*TAZ*/*TEAD* pathway. The selected genes (*TEAD1*, *WWTR1*) were verified in vitro and in mice. MRTX-849 treatment was also shown to induce a transcriptional program leading to increased integrin activation, cytoskeletal reorganization, actomyosin contractility, *RHO* activation, and consequent activation of the *YAP*/*TAZ* pathway. The use of *TEAD* inhibitors (1–4) has also been shown to significantly increase the efficacy of the *KRAS* G12C inhibitor in both in vivo and in vitro studies. The results presented above provide a landscape of potential targets for future combination strategies [53].

In the study by M. Pfeifer et al., genome-wide loss (CRISPRn) and gain (CRISPRa) of function were used to identify genes and pathways contributing to *EGFR*-dependent mechanisms of resistance to osimertinib in *EGFR*-mutated lung cancer [54]. This comprehensive functional genomics study of drug resistance in *EGFR*-mutated lung cancer revealed that the significant number of resistance genes converged on the Hippo pathway, which consists of a kinase cascade that regulates *TEAD*-dependent transcription through phosphorylation of the coactivators *YAP1* and *WWTR1 (TAZ)*. Inactivation of the Hippo pathway resulted in increased expression of the *YAP1*/*WWTR1*/*TEAD* genes. The expression of canonical transcriptional targets of the Hippo pathway was also examined after osimertinib treatment in 3D organoids and 2D cell line models. Ten xenografts from lung cancer patients with *EGFR* mutation (PDX) were used to characterize Hippo signaling after osimertinib treatment. In vitro as well as in vivo studies confirmed that *YAP1*/*WWTR1*/*TEAD*-dependent transcription is acutely activated after osimertinib treatment in *EGFR*-mutant lung cancer, and pharmacological and genetic ablation of this complex strongly inhibits persister cells. The study showed that activation of *YAP1*/*WWTR1*/*LEPR* dependent transcription is a potent factor of osimertinib resistance in *EGFR* mutant lung cancer [54]. In addition, acquired EMT status in cancer cells has been shown to prevent their death and lead to the formation of persistent cells. The presence of the EMT state has been detected in patients who develop acquired resistance to *EGFR* inhibitors. An alternative approach to prevent EMT transformation could be inhibition of *YAP1*/*WWTR1*/*TEAD*-dependent transcription.

In other studies, genome-wide CRISPR/Cas9 screening was performed on H460, H1299 and A549 cell lines that contain wild-type *EGFR* genes and identified the enzyme phosphoribosylaminoimidazole carboxylase/phosphoribosylaminoimidazole succinylcarboxamide synthetase (PAICS), which is essential for de novo purine biosynthesis and tumor growth, as a potential drug target for *EGFR* wild-type NSCLC [55]. PAICS protein levels were significantly elevated in tumor samples revealed by IHC staining in patients with adenocarcinoma. In vitro studies showed that *PAICS* knockdown only inhibited *EGFR* wild-type NSCLC carcinogenesis and induced cell cycle arrest. Among in vivo studies in mice, the authors confirmed that knockdown of *PAICS* significantly inhibited tumor growth. Moreover, *PAICS* deficiency promoted *EGFR* wild-type NSCLC apoptosis by inducing DNA damage both in vitro and in vivo. To summarize *PAICS*, as a new oncogene, may serve as a novel therapeutic target in *EGFR* wild-type NSCLC [55].

The CRISPR/Cas9 system was also used to generate specific research models. D. Maddalo et al. have developed an efficient method for inducing specific chromosomal rearrangements in vivo using viral delivery of the CRISPR/Cas9 system into adult animal somatic cells. This research approach enabled the creation of a mouse model of *Eml4-Alk*-induced lung cancer. The resulting tumors invariably contain *Eml4-Alk* invertase, express the *Eml4-Alk* fusion gene, and exhibit histopathological and molecular features typical of human ALK+ NSCLC. In addition, the tumors responded to treatment with ALK inhibitors so they show high potential as a research model [56].

CRISPR/Cas9 technology has also been used to introduce mutations in patient-derived cell lines for which it is difficult to obtain resistant variants due to the low incidence of cases in clinical settings. This approach has yielded a preclinical model of rare cancer subtypes such as ROS1+ NSCLC [57]. In the NSCLC cell line HCC78 with the SLC34A2-*ROS1* rearrangement, three *ROS1* mutations causing drug resistance, G2032R, L2026M and S1986Y, were knocked down using CRISPR/Cas9 technology. In addition, pharmacological assays in 2D and 3D cultures were prepared to evaluate the cellular response of the mutant lines. Spheroids derived from ROS1G2032R cells were significantly more resistant to repotrectinib, lorlatinib, ceritinib and entrectinib than wild-type cells. In conclusion, the study conducted on 3D cancer cell lines showed that lorlatinib and repotrectinib are the most effective TKIs against the tested *ROS1* resistance point mutations [57].

## 7. Chimeric Antigen Receptor CAR-T Cell Therapy in NSCLC

CAR-T cell therapy has revolutionized the treatment of hematologic malignancies, but unfortunately remains at an early stage for solid tumors such as NSCLC [58]. Key obstacles to the use of this treatment method include tumor heterogeneity, an immunosuppressive microenvironment and off-target toxicity. The most important aspects of using CAR-T therapy in NSCLC are described below.

Target antigens tested for CAR-T therapy in lung cancer include: *EGFR*, *MSLN*, *MUC1*, *CEA*, *PD-L1*, *ROR1*, *HHLA2*, *PSCA* and *HER2* [58]. *EGFR* is overexpressed in a significant percentage of NSCLC cases. In the study by Feng et al. patients with EGFR-positive (>50% expression), relapsed/refractory NSCLC received escalated doses of EGFR-targeted CAR-T cell infusions [59]. All patients (N = 11) had at least one recurrent (one patient)/metastatic or refractory lesion. The treatment of EGFR-targeted CAR-T cells were generally well tolerated. Of the 11 patients evaluated, two patients achieved a partial response and five had stable disease for two to eight months. Immunohistochemical examination (pre- and post-treatment biopsies) of tumor tissues/metastases after CAR-T cell infusion showed enrichment of CD3 + cells and reduction of EGFR + tumor cells, suggesting that anti-EGFR CAR-T cells may have reached tumor tissues and may be associated with elimination of EGFR-expressing tumor cells. The presence of the CAR-EGFR construct was confirmed in T cells infiltrating the tumor in four patients [59].

Other early-phase clinical trials have also investigated the use of EFGR-based CAR-T cell therapy to treat lung cancer [60]. In phase I clinical trial, nine patients with recurrent/refractory NSCLC and *EGFR* positivity received two cycles of the piggyBac-generated EGFR-CAR T cells therapy and showed good tolerance to treatment. The most common adverse event was fever. After treatment, one patient had a partial response of more than 13 months, while six patients had stable disease and two experienced disease progression. Progression-free survival in these nine patients was 7.13 months, while median overall survival was 15.63 months. This study revealed that the non-viral piggyBac transposon system-engineered EGFR-CAR T-cell therapy is feasible and safe in treatment of EGFR-positive advanced NSCLC patients [60].

The next target antigen tested for CAR-T therapy in NSCLC was mesothelin (*MSLN*) which expression is elevated in 69% of adenocarcinoma patients [61]. After stimulation with mesothelin, lentivirus-transduced T cells were stimulated to proliferate, express the anti-apoptotic gene *Bcl-X(L)* and secrete multiple cytokines. In in vivo studies in mice, the modified T lymphocytes reduced the tumor burden and in some cases caused complete eradication of tumors [62]. In the study of L. Ye et al. the second generation CAR-T cells were designed to target MSLN, which is abundant in NSCLC but underexpressed in normal tissues [63]. The second-generation construct contained co-stimulatory CD28 or 4-1BB signaling domains to enhance proliferation. CAR-T *MSLN* cells showed a significantly greater ability to kill tumor cells than T cells. In in vivo experiments, significantly slower tumor growth was observed in mice injected with CAR-T MSLN cells [63].

*MUC1* expression is abnormally elevated in NSCLC [64]. X. Wei et al. proposed investigated the antitumor potential of prostate stem cell antigen (PSCA) CAR-T cells and mucin 1 (*MUC1*) CAR-T cells in NSCLC [65]. Using PDX models that retained the antigenic profiles of the primary tumors, it was shown that PSCA-targeted CAR-T cells could effectively inhibit NSCLC tumor growth in PDX mice and synergistically eradicate PSCA + MUC1 + tumors in combination with MUC1-targeted CAR-T cells [65]. The study showed superior efficacy against NSCLC compared to treatment with CAR-T cells targeting a single antigen.

L. Wallsrabe et al. proposed the use of a 3D tumor model to investigate the anti-tumor effect of orphan receptor tyrosine kinase type 1 (ROR1-specific) CAR-T cells [66]. 3D tumors were created from the A549 cell line on a biological scaffold with a basement membrane, resulting in a progressive increase in cell mass and an invasive growth phenotype. CAR-T cells were shown to actively enter, adhere and infiltrate the tumor mass. Most importantly, it was confirmed that ROR1-CAR-T cells penetrated deep into the tumor tissue and eliminated multiple layers of tumor cells. Not only was the efficacy of the therapy confirmed, but also the utility of 3D models to investigate the anti-tumor function of CAR-T cells [66].

HER2-targeted CAR-T cells are a good therapeutic option in patients with HER2-positive NSCLC. However, a patient with colon cancer giving lung metastasis showed respiratory disturbances after infusion of HER2-targeted CAR-T [67]. It is assumed that this was related to low levels of HER2 expression on normal lung epithelial cells, which may have triggered an autoimmune response [68]. Therefore, the safety and efficacy of HER2-targeted CAR-Ts requires further investigation and the design of alternative antigen-specific targets. The team of L. Nagy, engineered UniCAR-T cells by incorporating the biotin-binding domain of monomeric streptavidin 2 (mSA2) to target HER2 via biotinylated trastuzumab (BT) [69]. Studies in 3D cultures and in vivo on a HER2+ xenograft model showed that in the presence of BT, UniCAR-T cells effectively penetrated HER2+ spheroids and targeted effector cells to HER2+ tumors. This therapeutic approach has been used for breast cancer however, it appears to be a good alternative for NSCLC as well.

One factor that decreases the efficacy of CAR-T cells is the immunosuppressive microenvironment of solid tumors. It is well known that increased expression of PD-L1 (programmed death ligand 1) may be a key factor contributing to the immunosuppressive TME in NSCLC. PD-L1 binds to PD-1 receptors on T lymphocytes, leading to inhibition of T lymphocyte activation and proliferation, reduced cytokine production, and exhaustion of cytotoxic T lymphocytes [70]. Previous studies have shown high expression of PD-L1 in tumor cells from NSCLC patients, and CAR-T cells secreting an anti-PD-L1 antibody have shown promising efficacy in humanized mouse models [71]. In the M. Liu et al. study, PD-L1-CAR-T cells have significant antitumor activity in vitro and lead to prolonged remission in xenografts of PD-L1-positive NSCLC tumors in mice [72]. Additionally, combining these CAR-T cells with subtherapeutic doses of local radiotherapy enhanced their efficacy against PD-L1 low NSCLC cells and tumors. These findings support preclinical support for targeting PD-L1 with CAR-T cells for the treatment of NSCLC and potentially other types of solid tumors [72].

In a study of Q. Liao et al., a novel dual-targeted CAR was constructed. A first generation CD19/HER2 CAR with a CD3ζ signaling domain was fused to a PD-L1 CCR containing a CD28 costimulatory domain [73]. The dual-targeted CARs were tested in vitro (A549 NSCLC cell line) while their safety and therapeutic efficacy were evaluated using an in vivo mouse model of human tumor xenografts. Dual-purpose CAR-T cells demonstrated similar cytotoxic activity against CD19/HER2+ tumor cells regardless of PD-L1, but enhanced cytokine release and improved proliferative capacity were only observed in the presence of both CD19/HER2 and PD-L1. Furthermore, CAR-T cells preferably destroyed tumor cells in xenografts both CD19/HER2 and PD-L1 positive. This finding demonstrates that PD-L1 can be used as a universal target antigen. Dual-targeted CAR-T cells can be used to reduce the risk of off-target toxicity while maintaining their potent antitumor efficacy in the treatment of PD-L1-positive solid tumors [73]. To the best of our knowledge, dual CD19/HER2 CAR-T cell therapies are not currently under clinical trial for the treatment of NSCLC, but HER2-targeted CAR-T therapies remain an area of active research in this disease. In addition, several clinical trials are investigating HER2-targeted CAR-T therapies in solid tumors. Anti-HER2 CAR-T cells with 4-1BB and CD3ζ signaling domains and have begun a phase I/II trial to demonstrate safety and feasibility in the treatment of HER2-positive solid tumors, including NSCLC (NCT01935843) [74]. In this study, anti-HER2 CAR-T cells would be administered for three days to patients with unacceptable toxicity. Another phase I/II clinical trial of CAR-T cells targeting HER2-positive tumors, among other NSCLC, was discontinued for safety reasons (NCT02713984) [74]. As a result, no clinical results have been reported to date for anti-HER2 CAR-T cell therapy in NSCLC.

Recent reports have implicated the use of anti-PD-L1-expressing inhaled nanoparticles loaded with the STING agonist cGAMP (aPD-L1 NVs@cGAMP), demonstrating increased CAR-T cell activity in models of orthotopic lung cancer and lung metastasis [75]. Nanoparticles were shown to accumulate in the lung and deliver STING agonists (the interferon IFN gene stimulator STING pathway) to cells with PD-L1 overexpression via PD-1/PD-L1 interaction. A reduction in the number of immunosuppressive cells in the microenvironment was demonstrated. Studies in an orthotopic lung cancer and lung metastatic model that combination therapy with CAR-T cells and aPD-L1 NVs@cGAMP strongly inhibits tumor growth and prevents recurrence. The above studies indicated the potential use of aPD-L1 NVs@cGAMP nanoparticles as an effective CAR-T cell enhancer to improve the efficacy of CAR-T cells against NSCLC tumors. The strategy of remodeling the lung tumor microenvironment may be very effective in improving the efficacy of CAR-T cells against solid tumors. A phase I clinical trial investigated the use of CAR-T cells targeting PD-L1 in patients with NSCLC and other solid tumors. The novel PD-L1 CAR (MC9999) using our humanized anti-PD-L1 monoclonal antibody was designed to simultaneously target tumor cells and immunosuppressive cells. The antigen-specific antitumor activity of MC9999 CAR-T cells was consistently observed in four solid tumor models: breast cancer, lung cancer, melanoma and glioblastoma multiforme [76]. PD-L1 CAR-T cells generated from the existing monoclonal antibody atezolizumab or from novel monoclonal/humanized antibodies targeting PD-L1 have shown promising preclinical results [72]. However, a phase 1 clinical trial testing PD-L1 CAR-T cell therapy in NSCLC resulted in a serious adverse event of pulmonary toxicity that occurred 47 days after infusion of CAR-T cells [77]. These data in the clinical trial suggest that PD-L1-targeted CAR-T cell therapies may potentially have off-target effects that require additional study.

Carcinoembryonic antigen (CEA) was also considered as promising target for CAR-T cell therapy in NSCLC, given its overexpression in approximately 70% of NSCLC cases and minimal presence in normal adult tissues [78]. Preclinical studies have demonstrated that that abnormal serum CEA levels were strongly correlated with increased whole-body metastatic potential in advanced NSCLC [79]. Study of L. Wang et al. [80], in which T cells expressing CAR specific for CEA were transferred into CEA-tumor transgenic mice that physiologically expressed CEA as their own antigen. Adoptive transfer mediated significant tumor regression, but resulted in weight loss, probably due to symptoms of mild cytokine release syndrome. In conclusion, the results suggest that adoptive therapy based on CEA-specific CAR cells may be effective in patients with CEA-positive solid tumors, but require further modifications to CAR-T cells and preconditioning regimens [80]. X. Zhu et al. developed hypoxia-responsive CAR-T cells (5H1P-CEA CAR), which is activated in the hypoxic tumor microenvironment to induce CAR-T cells [81]. They confirmed that CAR-T cells, initially in a “resting” state after in vivo infusion, went into a “storing” state characterized by increased HIF1α expression in hypoxic tumors derived from colon cancer and NSCLC cell lines. The anti-tumor efficacy of 5H1P-CEA CAR-T cells was observed in tumor clearance in PDX models. Moreover, CAR-T cells responding to hypoxia maintained lower differentiation and showed increased oxidative metabolism and proliferation during culture, and demonstrated the ability to mitigate the negative effects of hypoxia on T-cell proliferation and metabolism. In addition, CAR-T 5H1P-CEA cells showed reduced T-cell depletion and a better T-cell phenotype in vivo. The possibility of restricting CAR expression to the hypoxic tumor microenvironment was demonstrated, which may help increase the efficacy and safety of CAR-T cells in solid tumors [81]. In phase I trial of hypoxia-responsive CEA CAR-T cell therapy, patients with advanced solid tumors, including NSCLC, showed sustained tumor remission for more than 5 months after high-dose CEA-CAR-T therapy. CAR-T cells showed robust expansion. The therapy had acceptable toxicity with either intravenous or intravenous infusion (NCT05396300). The promising antitumor potential of intravenous infusion and prolonged tumor remission in the high-dose group were confirmed. The results presented above clarified that CEA is an attractive tumor-associated antigen for targeted immunotherapy of NSCLC.

Among the selected therapeutic targets, studies of CEA CAR-T cells for the treatment of NSCLC are ongoing and have yielded promising preliminary results in both basic and preclinical medicine. The promising results have formed the basis for initiating phase I clinical trials to evaluate the safety, efficacy and maximum tolerated dose of anti-CEA CAR-T cell therapy in CEA-positive cancers, including NSCLC (NCT02349724, NCT04348643) and advanced lung cancer (NCT06768151, NCT06043466). The phase I/II EVEREST-1 trial (NCT05736731) is evaluating Tmod CAR-T cell therapy targeting CEA (A2B530) in patients with solid tumors expressing CEA and loss of HLA-A*02 heterozygosity, including NSCLS. The results of these studies will provide valuable insight into the feasibility and efficacy of CAR-T therapies targeting CEA in patients with NSCLC.

Recently, the potential of CAR-T/NK cell therapy targeting erythropoietin-producing hepatocellular carcinoma A2 (EphA2) has been design as an effective treatment for NSCLC with high EphA2 expression [82]. CAR-T/NK cells targeting EphA2 showed antitumor activity against NSCLC cell lines A549 and H460 in vitro and in vivo. EphA2 CAR-T cells showed higher killing efficiency of A549 cells and increased secretion of IFN-γ, TNF-α and granzyme B. In contrast, studies in xenograft mouse models confirmed that A549 tumor growth was suppressed and the number of cells infiltrating the tumor had elevated [82]. The proposed research approach is a promising therapeutic strategy for the treatment of NSCLC.

In conclusion, the efficacy of CAR-T cells in the treatment of solid tumors is limited due to various factors, such as the lack of specific antigens, the complexity of TME and the potential toxicity associated with treatment. CAR-T therapy of solid tumors remains a challenge. Even after identifying the target antigen for a solid tumor, the CAR-T cell must be able to reach the tumor site. There is still a need to improve CAR-T cell trafficking and entry into the tumor, as well as to promote better signaling, less depletion and memory phenotypes in solid tumors. Fourth-generation CARs with multiple costimulatory domains may be a solution to improve CAR T-cell function. Based on the available research results, it seems that one solution for treating solid tumors is to combine CAR-T cell therapy with checkpoint inhibitors, armored CARs and suppression of other inhibitory factors in TME.

## 8. Challenges in Gene Therapy for NSCLC

Despite significant progress in research, gene therapy for NSCLC faces several challenges that significantly hinder its implementation in clinical practice.

Both the method and the choice of delivery system for therapeutic genes to NSCLC cells remain problematic. Viral vectors are often limited by the host’s immune response, while nonviral delivery systems may be insufficiently efficient. In early studies of CRISPR/Cas9 for gene editing in primary T cells, Cas9 and gRNA were delivered by virus or electroporation. Both methods show low targeting efficiency, and DNA electroporation is also highly toxic to T cells [83]. In recent years, the electroporation method of Cas9 ribonucleoprotein (RNP), recombinant Cas9 and sgRNA complex has been successfully used to transfect activated human T cells [84]. These challenges limit the practical application of gene editing and given the relatively low efficiency of gene editing and the cost of the therapy, its availability is restricted. However, new developments in delivery systems based on nanoparticles and modified viral vectors may improve the efficacy of gene therapies.

Another problem is the heterogeneity of NSCLC tumors, which prevents the development of universal therapies. NSCLC tumors often exhibit significant genetic diversity, both within a single tumor and between different tumors. This heterogeneity can lead to varied responses to gene therapies and contribute to the development of resistance [85].

Co-occurring genomic alterations in oncogenic genes and tumor suppressor genes have become the mainstays of molecular diversity in NSCLC. Activating mutations in *KRAS*, for example, are the most common oncogenic factors and are accompanied by alterations in *p53*, *LKB1*, *KEAP1*, *ATM* and *RBM10*. For mutations of multiple genes, there are some combination therapies, including targeted therapies (inhibitors) in combination with standard treatment for NSCLC. To the best of our knowledge, gene-based therapies in combination with other treatment strategies have not been widely studied. However, these approaches may improve outcomes compared to monotherapy, especially when based on genomic profiling. For instance, the emergence of subclonal populations with distinct genetic alterations can undermine the efficacy of targeted treatments. A. Guernet et al. developed a highly complex CRISPR barcoding system that enables high-resolution tracking of single, specific tumor cells. This approach has been used to model different mechanisms of EGFR resistance in lung cancer cells. In details, CRISPR barcoding has been used to model intra-tumor heterogeneity and resistance mechanisms in NSCLC. This approach makes it possible to track rare subclones containing mutations, such as *EGFR* T790M and *KRAS* G12D, which are associated with resistance to tyrosine kinase inhibitors. By simulating these mutations, researchers can study clonal dynamics and test combination therapies targeting multiple mutations simultaneously. This allows for the identification of rare, pre-existing resistant subclones potentially involved in mechanisms of acquired therapy resistance [86].

Another obstacle to gene therapies is the immunosuppressive nature of the TME, which can impede the efficacy of gene therapies, especially those that rely on immune activation. Factors such as regulatory T cells, myeloid-derived suppressor cells, and inhibitory cytokines contribute to this suppressive environment [85]. As previously described, one of the factors that reduces the efficacy of CAR-T cells is the immunosuppressive microenvironment of solid tumors. It is hypothesized that, among others, PD-L1 expression may be a key factor contributing to the immunosuppressive TME in NSCLC [70]. The strategy of remodeling the TME has been developed by T. Zhu et al., and may prove to be very effective in improving the effectiveness of CAR-T cells in the fight against solid tumors [75]. In the case of CAR-T therapy, combining CAR-T cell therapy with other treatment methods, such as chemotherapy, radiotherapy or immunotherapy, offers promising prospects.

## 9. Conclusions

Gene therapy offers a potentially transformative approach to NSCLC, particularly for patients with advanced disease who have exhausted conventional treatments. Despite many challenges, ongoing research and clinical trials are showing promising results, suggesting that gene therapy may be part of a personalized cancer treatment in the future. Further research into novel delivery mechanisms, as well as integration of gene therapy with traditional therapies such as chemotherapy, radiotherapy, and immune checkpoint inhibitors, will be crucial to improving outcomes for patients with NSCLC. Further research is needed to realize the full potential of gene therapy in the treatment of NSCLC.

## Figures and Tables

**Table 1:** Driver Mutations in Adenocarcinoma.

Gene	Alteration	Frequency	Targeted Therapy
EGFR	Exon 19 del, L858R, T790M	~15–35% (Asian > Caucasian)	Osimertinib, gefitinib, erlotinib
KRAS	G12C and others	~25–30%	Sotorasib (for G12C)
ALK	EML4-ALK fusion	~3–7%	Alectinib, lorlatinib, crizotinib
ROS1	Fusion	~1–2%	Crizotinib, entrectinib
BRAF	V600E mutation	~1–3%	Dabrafenib + trametinib
MET	Exon 14 skipping, amplification	~3%	Capmatinib, tepotinib
HER2	Exon 20 insertion	~1–3%	Trastuzumab deruxtecan
RET	Fusions	~1–2%	Selpercatinib, pralsetinib
NTRK	Gene fusions	Rare (<1%)	Larotrectinib, entrectin

## Data Availability

Not applicable to this study.

## Abbreviations

Adp53	adenoviral p53 vector
ASO	antisense oligonucleotide therapies
BRAF	v-raf murine sarcoma viral oncogene homolog B
CEA	Carcinoembryonic antigen
CRISPR	Clustered Regularly-Interspaced Short Palindromic Repeats
c-ROS1	receptor tyrosine kinase
EPC1	enhancer of polycomb 1
ERBB2	otherwise HER2: human epidermal growth factor 2
hRT	hypofractionated radiotherapy
MET	receptor tyrosine kinase
MSLN	mesothelin
MUC1	mucin 1
NGR1	neuregulin-1
NPRL2	Nitrogen Permease Regulator-Like 2
NSCLC	Non-small cell lung cancer
NTRK1	neurotrophic receptor tyrosine kinase
PKC-α	Protein Kinase C-alpha prostate stem cell antigen
PSCA	phosphoribosylaminoimidazole carboxylase
PAICS	phosphoribosylaminoimidazole succinylcarboxamide synthetase
rAd-p53	recombinant adenoviral p53 gene
RNAi	including RNA interference
ROR1	receptor tyrosine kinase type 1
TKIs	tyrosine kinase inhibitors
TUSC2	Tumor Suppressor Candidate 2 gene
siRNAs	small interfering RNAs
STAT3	signal transducer and activator of transcription 3
STAT6	Signal transducer and activator of transcription 6
